# Facilitators and barriers to applying a national quality registry for quality improvement in stroke care

**DOI:** 10.1186/1472-6963-14-354

**Published:** 2014-08-27

**Authors:** Ann Catrine Eldh, Mio Fredriksson, Christina Halford, Lars Wallin, Tobias Dahlström, Sofie Vengberg, Ulrika Winblad

**Affiliations:** Department of Public Health and Caring Sciences, Uppsala University, Uppsala, Sweden; School of Education, Health, and Social Studies, Dalarna University, SE 791 88 Falun, Sweden

**Keywords:** Evidence based practice, Facilitation, National quality registry, Quality improvement

## Abstract

**Background:**

National quality registries (NQRs) purportedly facilitate quality improvement, while neither the extent nor the mechanisms of such a relationship are fully known. The aim of this case study is to describe the experiences of local stakeholders to determine those elements that facilitate and hinder clinical quality improvement in relation to participation in a well-known and established NQR on stroke in Sweden.

**Methods:**

A strategic sample was drawn of 8 hospitals in 4 county councils, representing a variety of settings and outcomes according to the NQR’s criteria. Semi-structured telephone interviews were conducted with 25 managers, physicians in charge of the Riks-Stroke, and registered nurses registering local data at the hospitals. Interviews, including aspects of barriers and facilitators within the NQR and the local context, were analysed with content analysis.

**Results:**

An NQR can provide vital aspects for facilitating evidence-based practice, for example, local data drawn from national guidelines which can be used for comparisons over time within the organisation or with other hospitals. Major effort is required to ensure that data entries are accurate and valid, and thus the trustworthiness of local data output competes with resources needed for everyday clinical stroke care and quality improvement initiatives. Local stakeholders with knowledge of and interest in both the medical area (in this case stroke) and quality improvement can apply the NQR data to effectively initiate, carry out, and evaluate quality improvement, if supported by managers and co-workers, a common stroke care process and an operational management system that embraces and engages with the NQR data.

**Conclusion:**

While quality registries are assumed to support adherence to evidence-based guidelines around the world, this study proposes that a NQR can facilitate improvement of care but neither the registry itself nor the reporting of data initiates quality improvement. Rather, the local and general evidence provided by the NQR must be considered relevant and must be applied in the local context. Further, the quality improvement process needs to be facilitated by stakeholders collaborating within and outside the context, who know how to initiate, perform, and evaluate quality improvement, and who have the resources to do so.

## Background

With initiatives to better assess adherence to evidence-based guidelines being proposed around the world (e.g. [1–3]), Sweden has been cited a role model with over 80 registries qualifying as national quality registries (NQRs), many of them having been long established [4]. The NQRs provide for individual-based data entries to be gathered on particular problems or diagnoses, treatment interventions and outcomes. The registries are operated by professionals while subsidised by national authorities and are believed to facilitate continuous quality improvement, cultivating effectiveness, and to even out differences in quality of care between health providers [4]. Although it is generally perceived that the NQRs facilitate quality improvement (e.g. [5]), neither the extent of nor the mechanisms of the relationship between NQRs and quality improvement are fully understood. While the extended experience of Sweden’s NQRs could be of international interest, few studies have investigated this issue.

By collecting and systematically analysing data in order to understand quality of care and identify areas of improvement, medical and hospital registries and the like have been endorsed as tools for improving health care quality [6]. Bridging the know-do gap is essential yet still a challenge for modern health care [7] and is vital for quality improvement programmes aiming to engage in evidence based practice (EBP) [8]. Quality improvement is proposed as the combined and unceasing efforts of stakeholders, such as healthcare professionals and patients, to make the changes that will lead to better patient outcomes, better system performance, and better professional development [9]. A number of theoretical frameworks have been suggested to guide the implementation of EBP, one of them being PARIHS, the acronym for Promoting Action on Research in Health Services [10]. The PARIHS framework suggests that successful implementation is signified by a process that considers the nature and type of *evidence*, the qualities of the *context* in which the evidence is being introduced, and the way the implementation process is *facilitated.* Specifically, the framework proposes that the facilitating of implementation efforts require “transformational leaders, features of learning organisations, and appropriate monitoring, evaluative, and feedback mechanisms” ([11], p. 1). Tentatively, the NQRs have the possibility to support this, through their processes of monitoring, evaluating, and reporting of data. Yet, participation in an NQR does not automatically or entirely explain if, how, and when, the presence of data, data reporting, and possible feedback of local and/or national data, prompts or sustains quality improvement in a local setting. Rather, there is a need for further knowledge of what end-users experience as the relationship between NQR and quality improvement and which attributes of that relationship contribute to establishing and maintaining quality improvement. The aim of this case study is to describe the experiences of local stakeholders to determine those elements that facilitate and hinder clinical quality improvement with regards to participation in a well-known and established national quality registry on stroke in Sweden.

## Methods

### Design

This study is part of a larger project, applying mixed methods in an exploratory design to investigate if and how NQRs promote quality improvement at micro (clinic/hospital), meso (regional), and macro (country) level. This study is the primary study, focusing on the experience of stakeholders at micro level, and the findings will later be trialed in a quantitative survey [12]. In this case study, we applied a qualitative design [12], employing semi-structured interviews analysed with content analysis [13].

### Sample

Primarily, we sought out an NQR with a long history of endurance and high reach; *Riks-Stroke* (The Swedish Stroke Register), launched in 1994, is one of the largest Swedish NQRs [14]. Since 1998, all hospitals providing acute stroke care participate in the registry, and today Riks-Stroke comprises 72 hospitals. Every year, 25,000 to 26,000 unique health care cases of stroke are reported in the registry, including the acute phase of stroke, and follow up at 3 and 12 months post stroke incident for each patient [15].

Riks-Stroke is controlled by a steering committee overseeing the quality of the registry and advising the manager of the registry on issues such as development. Further, the steering committee suggests and executes research studies based on registry data. A team of staff at the Riks-Stroke’s secretariat provides daily maintenance of the registry and support to managers and staff in the hospitals. Since its launch in the mid-1990s, Riks-Stroke has obtained funding from the Swedish Board of Health and Welfare and the Swedish Association of Local Authorities and Regions [15].

For this study, a strategic sample was drawn of eight hospitals in four county councils, representing different parts of the country, and evaluations of ‘good’ , ‘fair’ , and ‘limited’ outcomes in the registry in 2011, according to Riks-Stroke criteria.

### Riks-Stroke’ s criteria for assessing stroke units

- Coverage >85%
- Proportion follow-up 3 months post stroke
- Proportion treated in stroke unit
- Proportion with nonstop admission to stroke unit
- Proportion where swallow test performed
- Proportion with thrombolysis treatment
- Time from arrival to hospital to thrombolysis start
- Proportion with antithrombotic treatment at discharge after brain infarct
- Proportion treated with anticoagulant if atrial fibrillation and brain infarct, <80 years of age
- Proportion discharged from hospital with antihypertensive drugs
- Proportion content with help and support after discharge
- Proportion with follow-up out-patient visit with physician and/or registered nurse

For each hospital, local stakeholders were invited to participate in individual telephone interviews: if the hospital was organised into clinics and departments, both the head of the division and the head of the department where the stroke unit was situated were invited to take part, while in cases where there was no division level, only the head of department was approached and vice versa. Further, the physician in charge of the Riks-Stroke registry and the registered nurse (RN) in charge of registering the local data for Riks-Stroke were also invited.

### Procedure

For all eight hospitals, initial contact was made via phone or e-mail to acquire names and e-mail addresses for the above stakeholders. Subsequently, a letter of information about the study was sent to each stakeholder, including potential times for interviews, and the possibility to suggest other dates/hours. Reminders were sent by e-mail once a month. Stakeholders who had not responded after three reminders were contacted via telephone to attain whether the lack of response was due to errors in contact details, a wish not to take part, or if the interview needed to be postponed.

Interviews were guided by a semi-structured interview guide developed for this study, drawing on an extended literature review on NQRs and quality improvement, and on Riks-Stroke, in addition to conversations with a stroke expert not partaking in this study. The guide included queries on 1) the informant’s role in relation to Riks-Stroke, 2) data capture and registration procedures, 3) reports and feedback, 4) quality improvement in clinical practice, 5) collaboration, 6) pros and cons of the NQR, and 7) the accountability of Riks-Stroke. A shorter version including fewer probes was applied for the upper level management, that is, the heads of division and/or heads of departments, as they generally would have less detailed knowledge of how registries are managed.

To ensure flexibility in the interview schedule and thus increase participation, all interviews were performed over the phone. The interviews lasted to 45–60 minutes for the physicians and the RNs, and 15 minutes for the upper level managers, respectively. The interviews were recorded, comprising the entire discourse between interviewer and stakeholder, and later transcribed verbatim. All participants provided informed consent to participation prior to the interviews, and approval of the study was obtained from the Research Ethics Board in Uppsala, Sweden (no. 2013/181).

### Data analysis

Initially, the corresponding author read and re-read the interview texts, becoming familiar with what the stakeholders shared. The focus was placed on capturing what signified barriers and facilitators within the context, including both observable aspects, such as the physical environment and availability of information resources, and underlying aspects, such as social interactions and management. Further, perceived barriers and facilitators within what is specified as “evidence” in the PARIHS framework were captured, encompassing sources of knowledge, such as research evidence, clinical experience, patient preferences and experiences, and local information. In addition, facilitation, that is to say, a process for translating research evidence into practice, and barriers and enablers of this process were identified, as advised in the PARIHS framework [16]. The naïve understanding of each interview and all the interviews together were brought together to illustrate “a sense of the whole” [13].

Subsequently, five of the transcribed interviews were analysed in detail, providing a matrix with categories clustered in relation to ‘context’ , ‘evidence’ , and ‘facilitation’ , respectively. Consequently, a structured analysis of all interviews was performed, applying and cultivating the matrix (as outlined in Table 1) [13].

**Table 1:** **Categories of context, facilitation, and evidence in the analysis matrix**

Factors suggested by the PARIHS framework to influence implementation of EBP	Categories identified and included in the structured analysis matrix
Context	Staff working with Riks-Stroke at the stroke unit
	Stakeholder competence
	Collaboration
	The stroke process
	The health care structure
	Local knowledge exchange
Evidence	The NQR/Riks-Stroke data
	National stroke guidelines
	Regional/local stroke guidelines
Facilitation	The NQR/Riks-Stroke
	Riks-Stroke staff
	External knowledge exchange
	Management system
	Process management
	Benchmarking

To finalise, categories and their corresponding content were abstracted and concluded, suggesting a comprehensive understanding [13]. Trustworthiness in the analysis was procured by recurrent dialogues within the research team with regards to the most valid understanding of the data and the rigor of the analysis [17].

## Results

Altogether, 25 interviews with local stakeholders were performed between March and September 2013. All invited stakeholders took part in the study, except one. The number of participants per stakeholder group is presented in Table 2.

**Table 2:** **Number of informants and stakeholder role at the eight hospitals included in the study**

Stakeholder role	Number of persons partaking in study interviews
Head of hospital division (if any) under which the department responsible for stroke patient care is located	3
Head of department (if any) responsible for stroke patient care	7
Physician in charge of Riks-Stroke	7
RN in charge of registration in Riks-Stroke	8

The findings at the structured analysis level are presented in relation to how they informed the elements of “context”, “evidence”, and “facilitation”, and are illustrated with quotes from interviews. To conclude, we will conceptualise those elements that stakeholders’ experience designates facilitate and hinder quality improvement in relation to the NQR Riks-Stroke and/or the health care context.

### Context

Among ***the staff*** working with the Riks-Stroke, a key person was the registered nurse (RN) in charge of the data registration. For the data to be valid, this RN needed to perform an ongoing, meticulous checking of the local data reported in Riks-Stroke. For the NQR to support quality improvement, the contributions of an RN with a keen interest in and knowledge of the stroke process, and stroke itself, was crucial.

*And there was no order until we had a person [RN] who was put in charge of it [the registration of data]. It’s a detailed process, validating the data.* (Physician, site 6)

Yet, this RN needed ***collaboration*** with and support from others; all staff on the stroke units, the physician in charge of the stroke registry in the hospital, and managers:

- The stroke unit staff entered the data in the electronic patient records (EPR) and other information systems later collated and entered in the NQR. The RNs in charge supervised the staff to record patient data in a way that would facilitate the NQR registration.
- While the RNs in charge of the Riks-Stroke registration spent much time tracking and checking the data, the validating process also involved the physician in charge, who needed to confirm diagnoses code entries in the patient records. Good collaboration between the RN in charge of registering and the physician in charge of the NQR secured the validity of data, and promoted local quality improvement initiatives.

All in all, ***the competence*** of the professionals in these key roles varied; the RN could be in charge of registration only or be an RN stroke specialist, the latter also being in charge of out-patient visits and post-stroke follow-ups. Further, the registration responsibilities varied, from registering only, to also collating and reporting the output data regularly or as and when requested. In addition, the physician could be a stroke expert, or an expert in another medical sub-specialty. He/she could be in charge of the unit’s registry in Riks-Stroke as well as being or not being in charge of the stroke process. The more dedicated the physician was to stroke and Riks-Stroke, the better the collaboration.

*Collaborating with a physician who’s not a stroke expert means no further dialogue on what can be improved [based on the Riks-Stroke data] in terms of quality of care.* (RN, site 4)

Although the engagement of the RN and the physician was considered to be crucial, managers also needed to be involved; the engagement of upper management stimulated the physician and nurse in charge of the local Riks-Stroke, particularly if the managers showed an interest in the output data. The RN and physician stakeholders generated interest in the data among staff and managers by presenting them in an informative and appealing format, illustrating evidence of improvements, and identifying issues of relevance to the everyday stroke care provided in the unit. If questioned or unsupported by others, the RNs and physicians experienced a decrease in their engagement in the NQR.

*One cannot present all statistics. One has to identify the trigger points, to pick the most interesting pieces of information. And compare that to previous years, yes - that’s the way to do it.* (RN, site 1)

A further context aspect was ***the stroke care process*** itself, which influenced the use of the NQR for quality improvement. While the scope of the data required by Riks-Stroke was considered to be extensive, and the process of assuring data validity highly demanding, staff working in hospitals where most patients with stroke were cared for in special stroke units described less work in making sure the data were accurate before entry in the NQR, than staff working in hospitals without special stroke units.

*All our [stroke] patients should be cared for in the stroke unit. While they are not, this is one of our goals, and we have to figure out how to reach it.* (Manager, site 8)

In addition, in hospitals where nursing staff and physicians were organized into separate ***health care structures***, the collaboration and flexibility needed for the NQR to induce quality improvement was stalled. Further, a lack of specialist nurses and physicians in stroke care meant more work went into verifying that the data were correct, and it was considered a negative influence on the possibility to provide a safe process of good quality of care for stroke patients.

*Like I was trying to cut the “door-to-needle” time, it wasn’t acceptable as it was. And I suggested four different ways of improving this part of the stroke path but none of the other organisations involved in the stroke process thought it to be their responsibility*. (Physician, site 3)

Moreover, ***local knowledge exchange***, that is, within the stroke unit or the hospital, was vital to the NQR work and quality improvement. In addition, managers considered the RN’s and physician’s engagement with regard to the stroke care and the NQR to be a key element, and the units’ main means for improving care.

*At one point, someone [staff] said “I can’t take any more negative feed-back”. “But it’s an improvement opportunity,” I said. So when we last met for planning, I could actually show how we had improved. (…) No, we don’t have any particular method for improvement; we discuss things [until we agree on what and how to do].* (RN, site 3)

Apart from local knowledge exchange and collaboration, ***stroke networks*** on a regional or national level were important; meeting others engaged in stroke and Riks-Stroke in regional networks was inspiring. Likewise, the annual NQR meetings, where updates on the Riks-Stroke and stroke knowledge, in addition to initiatives and results on quality improvementinitiatives, were shared, were useful for those attending.

*We thought we’d visit others [stroke units]. So we visited the xx, xy, and yy hospital and learned what they do (…). And we shared the ideas we had had and agreed on what and how to do. We brought it up with our management team, and started a project. And we went ahead and did it.* (RN, site 2)

### Evidence

Stakeholders considered ***the NQR Riks-stroke data*** to be a source of evidence. While the process of assuring validity in the registration of data was meticulous, it was considered crucial for producing valid and reliable output.

*I can, for example, check the number of thrombolysis we perform and compare to hospital yz and zz. And that’s a superb basis for innovations, and when presenting [to staff and managers], we can see if we are on track.* (RN, site 7)

Although the stakeholders described that the staff at the Riks-Stroke secretariat added new variables only after considering the value of additional output for the stroke units, they considered the Riks-Stroke registry to be at its maximum capacity in terms of number of variables. A direct transfer of data recorded by the medical, nursing, and rehabilitation staff, respectively, in the electronic patient record (EPR) to the Riks-Stroke data base was suggested, yet it was also questioned whether this approach was appropriate and possible in terms of ensuring the validity of the data entries. The stakeholders suggested that the stroke diagnosis itself hinders automatic data entry as it is based on information collected throughout and after the patient’s care episode. Rather, validity in the NQR required checking and sometimes making corrections before the data were registered in Riks-Stroke.

*And it’s extremely important that we have an NQR which is well supported [with regards to evidence], so that one gets reasonable output data from it, which one can present to stakeholders; it can be politicians, the staff, or patient organisations, and whoever should have a share of this information.* (Manager, site 4)

Stakeholders described the close relationship between the variables of Riks-Stroke and ***the national guidelines*** on stroke as an asset. Because many of the variables mirrored the evidence of the national guidelines, the output data for each hospital’s stroke unit became a source for local mapping of adherence to evidence.

*We are not completely compliant with the national guidelines, lacking some [professionals on the stroke team]. But as we have formed a stroke unit, we should be getting there.* (Manager, site 5)

Yet, although the national guidelines were viewed as relevant and appropriate, adaptation to the local context was considered necessary. Thus, the national stroke guidelines were translated into ***regional or local guidelines for stroke***. These guidelines were trusted to be congruent with the agreed evidence in the national guidelines, and further served as input to the assessment of whether local data mirrored evidence-based practice or not, and to what extent.

*As a manager, it’s a way of assessing what we do, in stroke care. And it helps us to retrieve [data on] how many patients with stroke we have. Even though we can access these data in other databases too, Riks-Stroke enhances them.* (Manager, site 6)

### Facilitation

***The NQR Riks-Stroke*** was perceived as facilitating local quality improvement by providing a picture of how well the unit adhered to evidence-based practice, as described above. Moreover, the collaboration of local stakeholders, particularly the RNs registering data in Riks-Stroke, along with the national ***Riks-Stroke staff****,* facilitated learning about the registry and about stroke care.

*Having access to the experience and knowledge of aa [an authority on the Riks-Stroke team] is valuable. Really.* (Physician, site 5)

The ***external knowledge exchange*** took place in networks around stroke and Riks-Stroke; the Riks-Stroke staff were available for those who contacted them, and provided good services. While the issues raised by the informants were mainly on registration of data and outcomes, reliable and prompt responses emphasized the trustworthiness of the NQR and its staff. Further, the trustworthiness of the NQR variables indicated to stakeholders that the NQR output data were reliable, and could be used to inform the ***local management system***.

*And we verify all [data] that we register, so that we get as complete and correct registration as possible. We use the [stroke] registry a lot. To check on areas where we need to improve.* (Physician, site 8)

***Process management*** of stroke care was frequently applied, often introduced by managers. Process management was linked to the Riks-Stroke in that it was either the output data that had initiated a revision of the stroke care process, or that the output data from Riks-Stroke were used to follow up on the process management, or a combination of these two approaches. Some stakeholders described the NQRs as part of the management system; those who did included Riks-Stroke as a facilitator for health care improvement, using the local data to inform managers on what was needed, and/or what to focus on in terms of present or future stroke care.

*Like, when there is a financial cutback, it’s easy to say: “Let’s skip the registration [in NQRs]”. Because it takes time. But that is not an option; we want to register because we believe it helps, in developing [the stroke care].* (Manager, site 1)

Above all, ***benchmarking*** was done. That is, stakeholders compared the local stroke data over time, or to equivalent stroke units known by the stakeholders to have reliable data entries. Although the national reports from Riks-Stroke were delayed, the RN stakeholders could access the local data and used these for reporting to fellow staff members, management, and media, when appropriate, as and when it was requested.

*It’s teamwork, and if you give the team something, you get more back. And what makes me happy is that when we reach such results [in the stroke process] it’s a joint effort in the team. And Riks-Stroke becomes the receipt [that we have achieved this].* (Physician, site 2)

### Facilitators and barriers in quality improvement applying Riks-Stroke

Four key aspects related to the context, to the NQR, and to local NQR activities were found to facilitate quality improvement:

1. Collaboration between the three stakeholders, i.e. the RN in charge of registering, the physician in charge of Riks-Stroke in the local hospital, and their manager. A mutual interest and dialogue on the needs for providing reliable and valid data, the local output data, and what this meant in terms of good quality of care, as well as areas of improvement facilitated quality improvement. In contrast, stakeholders working on their own conveyed that they made contributions to their units, yet, despite their efforts they did not reach as far in terms of quality improvement.
2. The Riks-Stroke registry’s variables, being drawn from the national guidelines on stroke, provided validity to and a purpose for the large number of registry variables. This helped in convincing staff to record patient data properly and thus helped the physician and RN stakeholders, respectively, to complete the reports on each patient in Riks-Stroke. Because of this link, the data collected and reported back signified local evidence and helped to identify needs for quality improvement, in addition to evaluation of local quality improvement outcomes.
3. Having resources for managing local data supports quality improvement. This included the stakeholders knowing and using the knowledge of: how data can feed into quality improvement initiatives; which data to retrieve from the NQR; how to translate the output data into pieces of information of interest to co-workers and managers; how to present data; and having forums for presenting local data. Where the NQR was perceived to stimulate quality improvement, the local data were compared with the outcomes of other stroke units known to have valid data, and/or the unit’s own past data, and/or to the goals of the stroke unit in terms of quality of stroke care. Further, resources included having opportunities to and knowing how to deal with identified local deficits in terms of quality of care, based on the Riks-Stroke data.
4. External collaboration. Collaborating within regional stroke process projects, networks on stroke care, and/or the Stroke care secretariat supported quality improvement in various ways. In particular, these forums provided opportunities for sharing ideas on improving stroke care and registration in the Riks-Stroke. To benchmark one’s unit in relation to other stroke units in the NQR contributed to quality improvement; primarily the RNs in charge of registration, but also the physician in charge of Riks-Stroke in the unit, used the NQR to identify units with superior stroke outcomes and contacted and learned from them.

Alternatively, a general barrier to quality improvement was the extensive local resources needed for registering the local data in the NQR. With the time and effort required to validate and register local data, resources for performing quality improvement could be stretched. For the NQR to promote quality improvement, the key stakeholders needed allocated time in addition to that devoted to the registration of data in Riks-Stroke.

**Figure 1:**
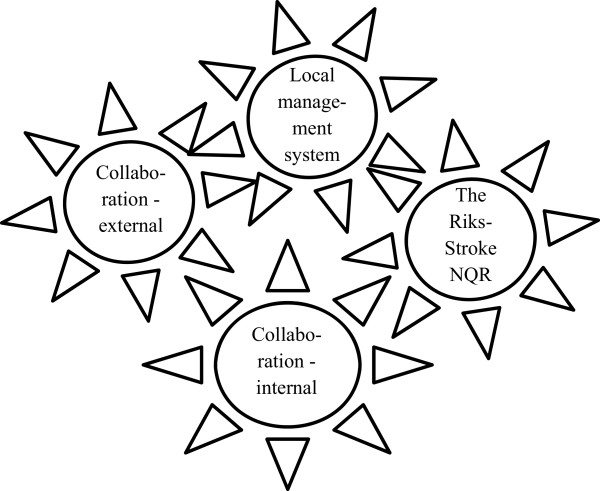
**The cogwheels related to the national quality registry facilitating continuous quality improvement– the Riks-Stroke example.**

Altogether, all four aspects contributed to the NQR progressing local quality improvement, while the stakeholders described this as an interaction; the more aspects in place, the larger the exchange. Enthusiastic and knowledgeable staff working with the registration and output data of the Riks-Stroke assured quality in the registration. Yet, if the data were to support quality improvement, there had to be a local team collaborating on the NQR, including managers and co-workers. All three stakeholders (that is, the RN, the physician, and the manager) needed to understand what the local data conveyed in terms of the quality of care and know how to improve care. While the staff at the national Riks-Stroke secretariat were available to support local initiatives, and there were forums where knowledge and experience was shared, it was imperative that the local stakeholders had access to and accessed this support. Further, if the local NQR was positioned within a local structure for managing quality of care, with resources allocated to perform quality improvement, the NQR was considered as only one yet a vital tool for stakeholders to identify needs for quality improvement, and to follow up improvement efforts. However, this required knowledge of how to initiate and sustain quality improvement in addition to knowledge on how to manage the in- and output of the NQR. These relations are further illustrated in Figure 1.

## Discussion

According to the literature, NQRs are most often but not always depicted as facilitating quality improvement (e.g. [18]) while there is limited knowledge of how and when this enablement occurs [19]. Although there are suggestions that separate quality improvement initiatives besides the NQR themselves are needed [20], others propose that the registration and output of data feed the plan-do-study-act of quality improvement itself [14]. In our study, the relationship between NQRs and quality improvement was found to be complex, suggesting that an NQR can contribute to quality improvement but does not automatically do so. Rather, the local context determines if and how the NQR induces quality improvement: according to stakeholders, quality improvement appeared where there was 1) collaboration among the local NQR stakeholders, and the collaboration included managers, and 2) contextual factors present, such as an active and purposeful management system supporting planning, performing, follow-up, and action on quality in the stroke care process, including the NQR. On the other hand, there were aspects connected to Riks-Stroke which supposedly hindered quality improvement, primarily the burden of data registration. With time being one of the constraints in clinical health care, data registration may occupy resources which could possibly be spent on improvement efforts [21].

Our findings suggest that stroke units’ stakeholders actively facilitated the NQR to allow managers and staff to grasp the local data, and also used the data to detect further needs for improvement and/or provided for understanding of how quality improvement efforts had influenced the stroke care. Although comparable units could be identified in Riks-Stroke, learning from others was an active strategy applied only by those who described Riks-Stroke as a factor contributing to the local quality improvement efforts. Stakeholders described that regional stroke networks were sources for engagement and learning, as were the staff at the NQR Riks-Stroke, and seminars arranged by the Riks-Stroke. Yet, not all stakeholders shared this learning experience. While earlier studies suggest that learning, in this case implementation of new knowledge, is a process related primarily to the individual [22], more recent studies suggest that context plays an important role [23]. As previously suggested in the PARIHS framework, implementation of evidence must function as a process combining the evidence, the context, and how implementation is facilitated [24]. Our findings suggest that when considering if and how stroke care data from an NQR facilitates EBP, again, the context in which the evidence is introduced, and the way the process is facilitated is fundamental. Participation in an NQR such as Riks-Stroke stimulates the quality improvement process by providing systematic data, and possibilities for comparisons with others [14]. In addition, including patient satisfaction on quality of care to some extent, Riks-Stroke provides information on patient preferences [14]. Thus, Riks-Stroke provides aspects of evidence such as local data corresponding to national guidelines and patient experience [24]. However, in this study, neither the reporting of local data nor having access to the evidence initiated quality improvement. Rather, quality improvement was initiated by people acting on the output data: stakeholders who know how to interpret the data and perform quality improvement [25].

A variation in stakeholders’ assignments and competence was identified; some, but not all, had knowledge on, as well as opportunities to carry out quality improvement initiatives. What caused this variation needs further investigation, considering contextual factors in the organisations and beyond. However, even stakeholders who had competence and opportunities to initiate quality improvement out of the local NQR data needed support by fellow staff and managers in their tasks. The relationship between NQR and quality improvement was considered opportune where there was a general interest in the data among staff and managers. Further, quality improvement required opportunities to share local data reports and to learn from one another, for NQR stakeholders as well as other professionals engaged in a particular care process such as stroke. While the managers relied on single staff to manage data entry and output assessments, one should recognise non-exhaustive structures and have processes in place to support job satisfaction among staff [26, 27]. In this study, barriers to using the NQR for quality improvement included a lack of the above factors, thus hindering optimal stroke care processes, collaboration, and knowledge exchange. Time to meet and discuss quality improvement is often limited in health care, and our findings emphasize that opportunities became even more restricted if the staff involved in stroke care were not organised in accordance with the stroke process. Managers were identified as key stakeholders in this study; likewise, leadership has been suggested as a major determinant of the working conditions, multidisciplinary involvement, and thus opportunities, for quality assurance [28].

Seemingly, our study, with its strategically chosen sample is of interest for decision makers, researchers, and clinicians who, use or suggest using NQRs as an assumed facilitator for quality improvement. The findings are supposedly transferable beyond Sweden and Swedish stroke care units, providing for an enhanced understanding of if and how NQRs promote quality improvement. Yet, we suggest additional studies to further investigate to what extent NQRs are means to improve quality of care, in what contexts, and what aspects within the registries and/or context facilitate the NQR-induced quality improvement process in various NQRs and health care contexts. In particular, the relations between higher level management and government of health care organisations and the structures and processes for quality improvement are of interest.

In this study, the PARIHS framework was applied [10]. The research team had extensive experience of applying PARIHS in implementation studies (e.g. [29–31]) and presumed that quality improvement, induced or not induced by a NQR, could relate to factors suggested as vital for implementing EBP. The flexibility of the framework, in addition to the acknowledgement of unpredictable aspects of context and facilitation [32], further encourages testing its potential on a research question like ours. Our findings emphasise the role of facilitation and context, respectively, in implementing EBP [33] signifying that the framework was suitable. We found that employing Riks-Stroke in local quality improvement, stakeholders as individuals and teams is vital, facilitating trustworthiness in the data input and outtake as well as initiating quality improvement [34]. Further, the context in terms of management and staff engagement, and its relation to the facilitation of quality improvement, also corresponds to key elements of PARIHS. However, the framework was applied on a generic level and the lack of conceptual clarity [32] was inclined to effect a lack of guidance whether or not PARIHS could illuminate details on if and how the NQR facilitated implementation of evidence, and in what context and by which means. Rather, the categories identified in structured analysis overlap to factors described earlier in PARIHS to some extent but not comprehensively. Most importantly, the identification of a NQR as a context, evidence, and/or facilitating aspect needs further exploration, along with additional studies on the stakeholders’ role and function. While our study indicates that the framework is applicable for investigating if and how a NQR facilitates quality improvement and/or EBP [33], it may support both the need for further development of PARIHS [35, 36] and exploring other theoretical frameworks and models on NQRs role in promoting EBP [37].

## Conclusion

While NQRs are presumed to facilitate quality improvement, this study provides for a more complex correlation: the NQR Riks-Stroke as a case provides aspects vital for facilitating implementation of evidence such as local data. However, the NQR itself does not initiate quality improvement but it is local stakeholders in charge of the registry, registering data, and/or managing the stroke unit who do. With all stakeholders operating in collaboration to improve quality of care, and including the NQR in this process, quality improvement may occur. Yet, the NQR needs to be incorporated in an operational management system to advance quality improvement in clinical stroke care practice, acting as a source of local evidence.
